# *Bifidobacterium breve* BB05 alleviates depressive symptoms in mice via the AKT/mTOR pathway

**DOI:** 10.3389/fnut.2025.1529566

**Published:** 2025-01-30

**Authors:** Yanni Pan, Qingling Huang, Yuan Liang, Yuwuqi Xie, Fang Tan, Xingyao Long

**Affiliations:** ^1^Chongqing Engineering Laboratory for Research and Development of Functional Food, Collaborative Innovation Center for Child Nutrition and Health Development, Chongqing Engineering Research Center of Functional Food, Chongqing University of Education, Chongqing, China; ^2^Department of Sleep and Psychology, The Fifth People's Hospital of Chongqing, Chongqing, China; ^3^School of Biological and Chemical Engineering, Chongqing University of Education, Chongqing, China; ^4^College of Pre-School, Chongqing University of Education, Chongqing, China

**Keywords:** *Bifidobacterium breve*, depressive symptoms, probiotics, chronic unpredictable mild stress, AKT/mTOR pathway

## Abstract

**Introduction:**

This study investigates the potential of *Bifidobacterium breve* BB05 (BB05) in mitigating depressive symptoms in a mouse model of Chronic Unpredictable Mild Stress (CUMS), with fluoxetine as a positive control.

**Methods and Results:**

High-dose BB05 (1.0 × 10^9^ CFU/kg, BB05H) significantly reduced anxiety- and depression-like behaviors in CUMS mice, as measured by the open field test, tail suspension test, and forced swim test. BB05 treatment also reduced pathological ileal damage, alleviated inflammation, and lowered serum levels of pro-inflammatory cytokines IL-6 and TNF-α. Additionally, BB05 increased serum 5-HT levels and decreased ACTH concentrations. Mechanistic analysis revealed that BB05 exerts antidepressant effects by activating the AKT/mTOR signaling pathway in the prefrontal cortex, promoting neuroprotection, neurogenesis, and synaptic plasticity.

**Discussion:**

These findings suggest that BB05, particularly at higher doses, effectively alleviates CUMS-induced depressive behaviors and improves physiological outcomes, supporting the use of probiotics as a potential treatment for depression by targeting the AKT/mTOR pathway.

## 1 Introduction

### 1.1 Overview of depression

Depression, a mental disorder primarily characterized by persistent low mood, diminished interest, and lack of pleasure (1). It is associated with high prevalence, recurrence, and significant disability. Although full remission can occur, many patients continue to experience residual symptoms (2). It is worth noting that major depression and other forms of depression need to be distinguished. Major depression is characterized by profound and lasting low mood, almost complete loss of interest in activities, etc., often accompanied by serious disorders in sleep, appetite, cognition, self-blame, suicidal thoughts or behaviors, and requires comprehensive treatment of drugs, psychology, and electroconvulsive therapy (3). Other forms of depression, such as mild depression, have relatively milder symptoms, such as intermittent bad mood and slightly reduced interest. They can be managed through psychological counseling and lifestyle adjustments such as regular work and rest, moderate exercise, etc. (4). Seasonal depression often occurs in specific seasons (such as winter) or is related to reduced sunlight. In addition to conventional antidepressant methods, light therapy can be used as an auxiliary treatment (5). Depression substantially impairs quality of life and negatively affects both mental and physical health. In recent years, the global incidence of depression has risen sharply, making it one of the most common mental illnesses and a major public health concern (6). Thus, the prevention, alleviation, and treatment of depression have become urgent global priorities.

### 1.2 Application of animal models in depression research

Animal models are widely used in preclinical research to explore potential treatments and prevention strategies for depression. Among these, the Chronic Unpredictable Mild Stress (CUMS) model, which simulates long-term stress and daily life stimuli, is ideal for studying depression mechanisms and assessing treatment efficacy (7). The CUMS model is of great significance for studying the effects of probiotics. In real life, when people are under chronic stress for a long time, the balance of the intestinal microbial community is often broken, which may affect the neuropsychiatric state and induce depression (8). The mechanism of action of probiotics is to regulate the intestinal microbiome (9, 10). The CUMS model can first induce intestinal and neuropsychiatric changes similar to those caused by chronic stress in humans. On this basis, the introduction of probiotic intervention can clearly observe how probiotics repair and adjust the unbalanced intestinal microbial ecology, and how this adjustment further affects the behavioral and neurobiological indicators related to depression, so as to deeply explore the action path and effect of probiotics in the prevention and treatment of depression. Behavioral experiments effectively evaluate the success of CUMS modeling and the impact of interventions, drawing considerable attention from the scientific community (11).

### 1.3 Exploration of the pathogenesis of depression and its potential association with *Bifidobacterium breve* BB05

The pathogenesis of depression is complex and remains incompletely understood. Recent studies suggest that impaired synaptic plasticity plays a critical role in the development of depression and related cognitive impairments, including deficits in learning and memory (12). The brain-derived neurotrophic factor (BDNF)/tyrosine receptor kinase B (TrkB) signaling pathway is essential for regulating synaptic plasticity and neuronal growth (13). In the context of the gut microbiota, especially with probiotics, there is an emerging interaction. The gut microbiota can influence the BDNF/TrkB pathway (14). For example, a healthy gut microbial balance may support proper BDNF expression and function, which in turn affects synaptic plasticity (15). Activation of this pathway leads to neuroprotection via the downstream serine/threonine kinase (AKT)/mechanistic target of rapamycin (mTOR) signaling cascade, which also triggers anti-apoptotic mechanisms that mitigate depressive behaviors (16). Previous research has shown that ketamine enhances glutamate transmission, activates the AKT and extracellular signal-regulated kinase (ERK) pathways, stimulates mTOR-mediated synaptogenesis, and increases BDNF and TrkB levels, producing neuroprotective, neurogenic, and neuroplastic effects (17). The mTOR pathway, has an important influence on depressive behavior (18), plays a critical neuroprotective role by regulating autophagy, promoting protein synthesis, and inducing neural regeneration (19). It forms two distinct complexes, mTORC1 and mTORC2. mTORC1 is vital for neuronal development, neurogenesis, and synaptic plasticity, while mTORC2 promotes cell survival and cytoskeletal organization (20). Additionally, the transcriptional coactivator peroxisome proliferator-activated receptor gamma coactivator-1 alpha (PGC-1α) facilitates the degradation of the neurotoxic metabolite kynurenine, helping prevent stress-induced depression by regulating mitochondrial biogenesis and oxidative phosphorylation (21). The neurotransmitter serotonin (5-HT) also plays a key role in regulating mood, memory, cognition, appetite, and sleep. Its normal expression is crucial for preventing the onset of depression (22).

It is worth noting that *Bifidobacterium breve* BB05, as a potential probiotic strain, has been preliminarily found to intervene in depression-related mechanisms through a unique pathway. It may indirectly affect the synthesis and metabolism of neurotransmitters by regulating the composition and function of the intestinal microbial community, such as the regulation of 5-HT, and thus play a positive role in the signal transduction of the gut-brain axis. At the same time, BB05 may be able to affect the nutritional support system of neurons, produce synergistic effects with nerve growth factors such as BDNF, enhance the plasticity and stress resistance of neurons, and provide new perspectives and targets for the prevention and treatment of depression. Thus, targeting the AKT/mTOR pathway is essential for developing effective interventions for depression, and the potential regulatory role of *Bifidobacterium breve* BB05 in this complex network deserves further exploration.

### 1.4 Limitations of existing depression treatment measures and exploration of new directions

Current treatments for depression remain suboptimal. Antidepressant medications are often expensive, require prolonged use, have limited efficacy, and are associated with adverse side effects, particularly with long-term administration (23). According to relevant studies, about 30–50% of patients receiving antidepressant medication fail to achieve significant symptom relief, that is, the treatment inefficiency is high (24). At the same time, long-term use of antidepressants may cause a series of side effects, such as weight gain. About 20–30% of patients will experience weight gain to varying degrees (25); the incidence of sexual dysfunction can also reach about 15–40% (26); In addition, it may also lead to adverse reactions such as aggravation of sleep disorders and gastrointestinal discomfort (27). Consequently, there is an urgent need to identify effective, non-pharmacological interventions for depression. Both preclinical and clinical studies have shown that the gut microbiota plays a crucial role in the gut-brain axis, which is implicated in the pathogenesis of various neuropsychiatric disorders, including stress, autism, depression, and dementia (28). Probiotics, as beneficial microorganisms, help maintain a healthy gut environment by modulating the intestinal microbiota, supporting the gut-brain axis, and offering potential adjunctive therapeutic effects for neuropsychiatric disorders (29). Growing clinical evidence suggests that probiotics can regulate stress responses and alleviate emotional and anxiety symptoms in individuals with chronic fatigue and irritable bowel syndrome (29). For instance, *Lactobacillus* (*L.*) *paracasei* PS23 has been shown to reverse anxiety and depressive behaviors induced by long-term corticosterone exposure (30), while *L. fermentum* PS150 mitigates the reduction in serotonin levels, lowers corticosterone concentrations, and restores normal behavior in rats subjected to chronic mild stress (31). Additionally, *L. reuteri* NS8 has been found to improve behavioral and cognitive impairments induced by chronic stress in rats by reducing plasma corticosterone and adrenocorticotropic hormone levels, restoring hippocampal serotonin (5-HT) and norepinephrine levels, and upregulating BDNF gene expression (32). Furthermore, *Bifidobacterium breve* CCFM1025 alleviates severe depression by modulating the gut microbiota and tryptophan metabolism (33). The above previous research results indicate that probiotics can improve the structure of intestinal microbiota and regulate neurotransmitter levels, thereby alleviating the mood of patients with anxiety or depression. Perhaps this is also the potential mechanism of *Bifidobacterium breve* BB05 in alleviating depression.

In this study, we developed a CUMS depression model in mice and used fluoxetine as a positive control to assess the effects of *Bifidobacterium breve* BB05 (BB05) on depressive symptoms. We further explored the underlying mechanisms through behavioral assessments, including the open field test, tail suspension test, and forced swim test, in addition to molecular biology experiments. Fluoxetine was chosen as a positive control mainly based on its prominent position and wide application in the field of depression treatment (34). Fluoxetine, as a classic selective serotonin reuptake inhibitor (SSRI) (35), has been confirmed by a large number of clinical studies to effectively increase the extracellular concentration of serotonin in the brain, thereby exerting a positive regulatory effect on mood and significantly relieving Depressive symptoms (36). By setting it as a positive control, it can provide a reliable reference standard for evaluating the intervention effect of *Bifidobacterium breve* BB05 (BB05) in depression model mice.

## 2 Experimental materials and methods

### 2.1 Materials and reagents

Fluoxetine (Sigma-Aldrich LLC), sodium chloride (Shanghai Macklin Biochemical Co., Ltd.), 4% paraformaldehyde general tissue fixative (Biosharp Life Sciences), TRIzol reagent (Thermo Fisher Scientific Inc.), 5-HT Kit (Nanjing Jiancheng Bioengineering Institute), adrenocorticotropic hormone (ACTH), interleukin-6 (IL-6), tumor necrosis factor-α (TNF-α) ELISA kits (Shanghai Enzyme-linked Biotechnology Co., Ltd.), Revert aid first-strand cDNA synthesis kit, 5× gDNA digester mix, 4 × Hifair III SuperMix plus, RNase-Free Water, SYBR Green PCR Master Mix (Yeasen Biotechnology, Shanghai, Co., Ltd.), isopropanol and chloroform (Chengdu Kelon Chemical Reagent Factory).

### 2.2 Instruments and equipment

Upright Microscope 6D45415 (Olympus Corporation, Japan); Bioprep-24 Biological Sample Homogenizer (Hangzhou Allsheng Instruments Co., Ltd.); A200 Gradient PCR Machine (Hangzhou LongGene Scientific Instruments Co., Ltd.); Multifunctional Microplate Reader VLBL0TD1 and StepOnePlus Real-Time PCR System (Thermo Fisher Scientific (China) Co., Ltd.); LDZM Vertical Steam Sterilizer (Shanghai Shenan Medical Instrument Factory); Clean Bench (Suzhou Antai Air Technology Co., Ltd.); BY-G20 Medical Centrifuge (Beijing Baiyang Medical Equipment Co., Ltd.); HH-4 Digital Thermostatic Water Bath (Shanghai Jiangxing Instruments Co., Ltd.); BI-I50A Low-Temperature Biochemical Incubator (Steedtech Instrument Equipment (Shanghai) Co., Ltd.); AMR-100 Automated Microplate Analyzer (Hangzhou Allsheng Instruments Co., Ltd.).

### 2.3 Experimental strain

*Bifidobacterium breve* is a Gram-positive, non-motile, non-spore-forming anaerobic bacterium. The cells are diverse in morphology, usually short rods or clubs, about 0.5–1.5 μm × 1.5–8 μm in size, and arranged singly or in pairs or chains. Studies have found that *Bifidobacterium breve* can regulate nervous system function and improve anxiety-like behavior in mice by producing neurotransmitters or neuroactive substances, such as *γ*-aminobutyric acid (GABA) (37). *Bifidobacterium breve* BB05 (BB05), isolated from the breast milk of healthy adults in Zhangpu Town, Kunshan City (Jiangsu Province, China) was used in this experiment. It was preserved at the China General Microbiological Culture Collection Center (CGMCC; preservation number 24982). The strain was stored in a glycerol cryopreservation tube, inoculated in an MRS liquid medium at an inoculum of 2%, and incubated at 37°C for 16–24 h. After the strain grew in the liquid medium, a second activation was performed. A 2% sample of the bacterial solution was added to a new MRS liquid medium and incubated for 16–24 h at 37°C, then centrifuge and prepare bacterial suspensions of corresponding concentrations (1.0 × 10^7^ CFU/kg and 1.0 × 10^9^ CFU/kg) with saline.

### 2.4 Animal experiment design

In this experiment, 50 six-week-old SPF-grade male C57BL/6 mice were obtained from Hunan Slake Jingda Experimental Animal Co., Ltd. (License No.: SCXK [Hunan] 2019-0004). The mice were housed in the barrier system animal facility of the Collaborative Innovation Center for Child Nutrition and Health Development. The environmental conditions were maintained at 25 ± 2°C with 50 ± 5% humidity and a 12-h light–dark cycle. Food and water were provided *ad libitum*. The animal experiment protocol was approved by the Collaborative Innovation Center for Child Nutrition and Health Development (Animal Ethics Number: 2023032704B) and complied with the 2010/63/EU directive, during the experiment, we followed the principles of animal welfare and provided a comfortable environment and food. We adhered to reasonable and necessary design, reduced stress and pain, set a humane endpoint, terminated the experiment with compliant euthanasia methods, and ensured ethical standards.

After a one-week acclimatization period, all mice were weighed and numbered in sequence. The paper strips with the mouse numbers were randomly placed in the corresponding group containers by drawing lots until 10 paper strips were placed in each container, corresponding to 10 mice in each group, so as to achieve random grouping and make the selection of mice in each experimental group random. The mice were divided into Normal, Control, Fluoxetine (positive control, intraperitoneal injection of 0.2 mL 10 mg/kg fluoxetine), BB05 low concentration (BB05L, oral gavage 0.2 mL 1.0 × 10^7^ CFU/kg BB05 bacterial solution once a day), and BB05 high concentration (BB05H, oral gavage 0.2 mL 1.0 × 10^9^ CFU/kg BB05 bacterial solution once a day). All groups, except the normal group, underwent a five-week CUMS regimen as described in Table 1. Behavioral assessments, including the open field test, tail suspension test, and forced swim test, were conducted in the sixth week. In the seventh week, mice are anesthetized with chloral hydrate at a concentration of 4%, delivered by injection, and eye blood is taken after the mice enter deep anesthesia, and the mice were euthanized, and samples were collected for further analysis.

**Table 1:** Animal experiment grouping and treatment methods.

Group	Handing methods
Normal	Gavage with 0.2 mL of sterile normal saline
Control	CUMS + Gavage with 0.2 mL of sterile normal saline
Fluoxetine	CUMS + Intraperitoneal injection of 0.2 mL of 10 mg/kg fluoxetine
BB05L	CUMS + Gavage with 0.2 mL 1.0 × 10^7^ CFU/kg BB05 suspension
BB05H	CUMS + Gavage with 0.2 mL 1.0 × 10^9^ CFU/kg BB05 suspension

### 2.5 Establishing the CUMS animal model

The CUMS depression model was established following previously described protocols (38, 39). During the five-week modeling period (weeks 1–5), 10 different stress stimuli were randomly applied to the mice, ensuring that no stimulus was repeated for three consecutive days. The stimuli were as follows:

1. Tilted Cage: One end of the cage was elevated to create a 45° tilt, which was maintained for 24 h. It is expected that mice will find it difficult to maintain balance and comfort due to the tilt of the cage, and will move frequently to try to find a stable position, which may cause anxiety and increase activity. The purpose is to create a stressful environment by changing the angle of their living space.
2. Food Deprivation: Removal of food for 24 h. Mice will show a strong desire for food due to hunger, and may sniff around in the cage to find food and become restless. This stimulation is designed to simulate the stress caused by food shortage.
3. Water Deprivation + Empty Bottle Stimulus: Water is removed from the water bottle and then placed back empty for 24 h. Mice will constantly approach the water bottle to try to drink water. After finding that there is no water, they may gnaw the water bottle or show anxious behavior, which can trigger a stress response caused by thirst.
4. No Bedding: Removal of the bedding for 8 h. Mice lose their comfortable lying environment and may curl up in the corner, reduce activity and reduce sleep quality, so as to test their ability to adapt to changes in environmental comfort.
5. Continuous Lighting: Exposure to continuous light for 24 h. Disrupt the biological clock of mice, causing their sleep disorder, and may cause irregular day and night activities and restlessness. It is used to explore the impact of abnormal light on their physiological and psychological.
6. Crowding Stimulus: Insertion of partitions in the cage to reduce the activity area of the mice for 24 h. The activity space is limited, and mice may fight or collide with each other frequently due to competing for limited space, causing social stress and anxiety.
7. Tail Clamping: Clamping the tail 1 cm from the tip with a hemostat for 60 s. The instant pain will cause mice to struggle and scream, and then they may be afraid of the surrounding environment and hide in the corner for a long time. Evaluate the psychological impact of acute pain on them.
8. Day-Night Reversal: Adjusting the day-night cycle by covering the cage with a black cloth during the day and removing it at night, thereby creating a dark environment during the day and a bright environment at night. Similar to continuous light, it disrupts the biological clock, causing the behavior pattern of mice to be reversed, and they will be sleepy during the day, active at night, and in poor mental state.
9. Restraint: Restriction in a 50-mL plastic syringe for 2 h. When the body is restricted, mice will struggle and try to break free. After being released, they may panic and escape. Test their stress response to physical restraint.
10. Wet Bedding: Pouring 200 mL of water into the bedding for 8 h. The humid environment makes mice uncomfortable, and they will try to avoid wet places. They may shiver due to the cold, which increases their stress perception of adverse environments.

The success of the CUMS model is assessed by comparing physical signs and behavioral experiment results. Healthy mice typically display smooth, glossy fur and an alert mental state, while mice with depression-like symptoms exhibit dull, yellowish fur, lethargy, and significantly reduced grooming behavior.

### 2.6 Behavioral experiment

Behavioral testing is a crucial tool in rodent depression models, allowing researchers to predict depression progression and assess the depressive state of animals prior to molecular and cellular analyses. The testing methods employed in this study were based on protocols described by Tian et al. (38) and Antoniuk et al. (39).

Open Field Test (38): The open field test measures exploratory behavior and anxiety. The apparatus is a rectangular box (50 cm × 50 cm × 50 cm). At the start of the test, the mouse is placed at the center, and the observation begins immediately. The entire session is recorded on video for 6 min, and silence is maintained throughout. After each session, the apparatus is cleaned, and medical alcohol is used to disinfect and remove odors. Behavioral data, including total distance traveled, number of entries into the central area, and time spent in the center, are analyzed using ANY-maze 7.0 software.

Tail Suspension Test (38): In the tail suspension test, the distal end of the mouse’s tail, approximately 1.5 cm from the tip, is taped to a stand, suspending the mouse about 30 cm above the ground. The session is recorded on video for 6 min, with immobility time measured from the 3rd to the 6th minute. Immobility is defined as minimal movement, where the mouse’s body contour repeats at 82% within a 2-s interval, and data are analyzed using ANY-maze 7.0 software.

The forced swimming test (38) assesses depression-like behaviors in mice. Each mouse is placed in a transparent cylindrical glass tank (12 cm in diameter, 30 cm in water depth) filled with water at 23–25°C. A day prior to the test, mice undergo a 10-min acclimation session. In the formal experiment, each mouse swims for 6 min. Immobility time, defined as when the mouse remains still or only moves its hind limbs slightly, is recorded. After the test, the mice are immediately dried with a towel and warm air to prevent hypothermia.

In the open field test, the shorter the time the mice spent in the central area, the more serious their anxiety problems. In the tail suspension test and forced swimming test, the immobility time of mice was positively correlated with their despair. In addition, the order and interval of the above behavioral tests are usually arranged as follows: first, the open field test is carried out, usually 1–2 days after the completion of the CUMS model; the tail suspension test is carried out after an interval of 2–3 days; finally, the forced swimming test is carried out 3–4 days after the end of the tail suspension test.

### 2.7 Anatomy and sample collection

Mice were fasted for 12 h before dissection, but water was provided ad libitum. Whole blood was collected and centrifuged at 4°C at 4,000 rpm for 15 min. The supernatant serum was carefully aspirated, aliquoted (100 μL), and stored at −80°C for future analyses (Usually relevant indicators are tested within 1 month after sampling). The prefrontal cortex was dissected on ice, transferred into sterile centrifuge tubes, and stored at −80°C for subsequent investigations (Usually relevant indicators are tested within 1 month after sampling). Additionally, a 0.5 cm section of ileum, free of fecal matter, was excised and placed in a centrifuge tube containing 1.5 mL of tissue fixative (Usually staining is done within 2 weeks of sampling).

### 2.8 Observation of histopathological damage in ileal tissue

Ileum tissue sections were prepared, deparaffinized, and rehydrated with a graded ethanol series. After washing with distilled water, the sections were stained with hematoxylin for 20 min and rinsed under running tap water until they appeared blue. The sections were then immersed in 1% hydrochloric acid ethanol solution for 10 s, followed by another rinse with tap water. Subsequently, the sections were stained with eosin for 2 min. Post-staining, the tissues were dehydrated in high-concentration ethanol, cleared in xylene, and mounted with coverslips. Pathological changes in the ileal tissue were examined under a light microscope (Olympus BX43) using 10X and 20X magnifications.

### 2.9 Determination of the expression of relevant factors in the serum using ELISA

Serum levels of 5-HT (detection range 3.12–200 ng/mL), ACTH (detection range 1.00–2,000 pg./mL), IL-6 (detection range 1.56–100 pg./mL) and TNF-α (detection range 7.8–500 pg./mL) were determined using ELISA, following the manufacturer’s instructions for the assay kit.

### 2.10 RT-qPCR was used to determine the mRNA expression levels of relevant genes in the hippocampus and prefrontal cortex regions

Real-time quantitative reverse transcription polymerase chain reaction (RT-qPCR) was used to quantify mRNA expression levels in the hippocampus and prefrontal cortex. Total RNA was extracted from tissue samples using TRIzol Reagent, and its concentration and purity were measured. The RNA was then reverse-transcribed into complementary DNA (cDNA), followed by amplification via qPCR. The PCR cycle conditions included an initial denaturation at 95°C for 5 min, followed by 40 cycles of denaturation at 95°C for 10 s, and annealing/extension at 60°C for 30 s. The relative expression levels of target genes were calculated using the 2^−ΔΔCT^ method, with HPrT serving as the reference housekeeping gene. The primer sequences used in the experiment are listed in Table 2. These genes were selected because they play key roles in neural plasticity, signal transduction, energy metabolism, etc., and can reflect changes in molecular mechanisms related to stress in the prefrontal cortex.

**Table 2:** Primer sequences.

Gene	Primer sequences (5′-3′)
*HPrT*	F: CATCGCGCTAGACAGGTACTG R: CAATGAGCCAAGTGAGCGAGA
*mTOR*	F: GGCACACATTTGAAGAAGCAG R: CTCGTTGAGGATCAGCAAGG
*mTORC1*	F: TGCCCAACGTGAACCAGATT R: CCCATGATGTCGTGTGGTCC
*mTORC2*	F: ATGAACCCTAACCCCCAAGAC R: CGTTCTCCTCAATAGCAGGGA
*AKT*	F: AGAAGAGACGATGGACTTCCG R: TCAAACTCGTTCATGGTCACAC
*ERK*	F: GGTTGTTCCCAAATGCTGACT R: CAACTTCAATCCTCTTGTGAGGG
*PGC-1α*	F: TATGGAGTGACATAGAGTGTGCT R: CCACTTCAATCCACCCAGAAAG

### 2.11 Data analysis

Experimental results were expressed as mean ± standard deviation. The homogeneity of variances was evaluated using one-way ANOVA, performed with SPSS 22 software. Duncan’s multiple range test (*p* < 0.05) was used to determine significant differences, indicated by distinct letters in graphs and tables. Grubbs’ criterion was used to identify and handle outliers.

## 3 Results

### 3.1 The effect of BB05 on body weight changes in mice

**Figure 1:**
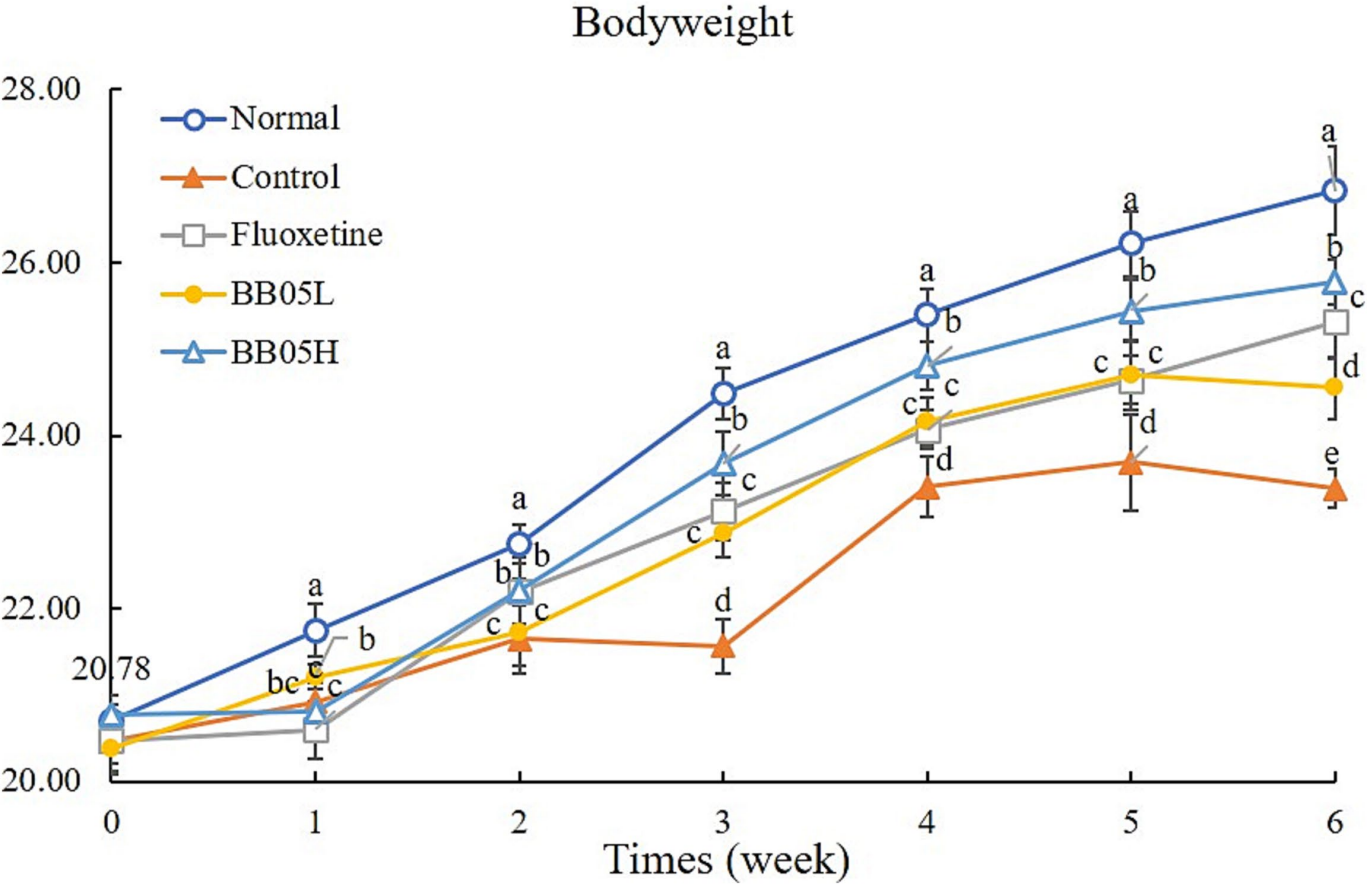
The effect of BB05 on the body weight changes in mice. Different lowercase letters indicate significant differences (*p* < 0.05).

As shown in Figure 1, no significant differences in body weight were observed among the groups before the formal experiment began. The trend in body weight gain remained stable during the 2 weeks preceding the induction of CUMS. However, 2 weeks after CUMS induction, the rate of body weight gain in the model, fluoxetine, BB05L, and BB05H groups decreased compared to the normal group, with significant differences observed among the groups (*p* < 0.05). From the third week onward, the body weight of mice in the model group significantly decreased (*p* < 0.05). By the end of the six-week induction period, the model group exhibited the lowest body weight, which was significantly different from the other groups (*p* < 0.05). Notably, the BB05H group displayed a significantly higher body weight compared to the BB05L and fluoxetine groups. These findings suggest that CUMS induction reduces the rate of body weight gain in mice, but interventions with fluoxetine and BB05 mitigated this effect. The BB05H group was more effective in maintaining normal body weight compared to the BB05L and fluoxetine groups.

### 3.2 BB05 alleviates anxiety and depressive-like behaviors in mice

**Figure 2:**
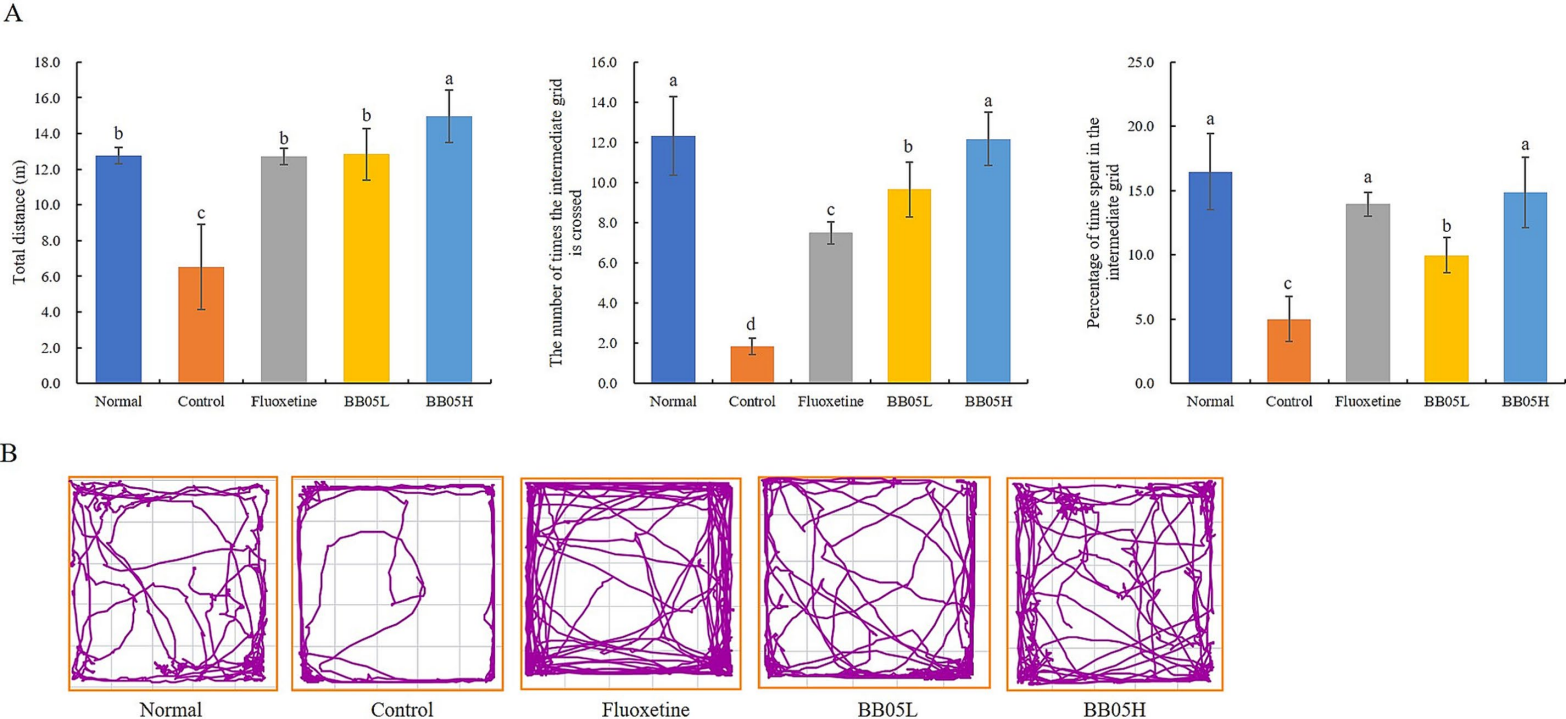
The effect of BB05 on the open field behavior in mice. **(A)** Results of the open field test; **(B)** Open field activity trajectory map. Different lowercase letters indicate significant differences (*p* < 0.05).

The open field test was used to assess anxiety-like behavior in rodents by measuring their exploration of new environments and aversion to brightly lit, open spaces. In this test, a shorter duration spent in the central area reflects higher anxiety levels. As shown in Figures 2A and B, mice in the model group traveled a total distance of 6.52 ± 2.38 meters, significantly lower than the other groups (*p* < 0.05). The number of crossings into the central squares was also significantly lower in the model group (1.83 ± 0.41 crossings) compared to the normal, fluoxetine, and BB05 groups (*p* < 0.05). Notably, the BB05 high-dose group (BB05H) exhibited a significantly higher frequency of crossings than both the fluoxetine and BB05 low-dose groups (BB05L), with no significant difference compared to the normal group (*p* > 0.05). Furthermore, the model group spent the lowest percentage of time in the central squares (5 ± 1.75%), which was significantly lower than the other groups (*p* < 0.05). Following treatment with fluoxetine and BB05, the time spent in the central area increased significantly. The BB05H group spent a higher percentage of time in the central area than both the fluoxetine and BB05L groups, with no significant difference from the normal group (*p* > 0.05).

These findings suggest that CUMS-induced stress heightened anxiety and significantly reduced activity levels in mice. However, after interventions with fluoxetine and BB05, activity levels significantly improved, approaching those of the normal group. Additionally, the BB05H group demonstrated a stronger regulatory effect than the BB05L group.

**Figure 3:**
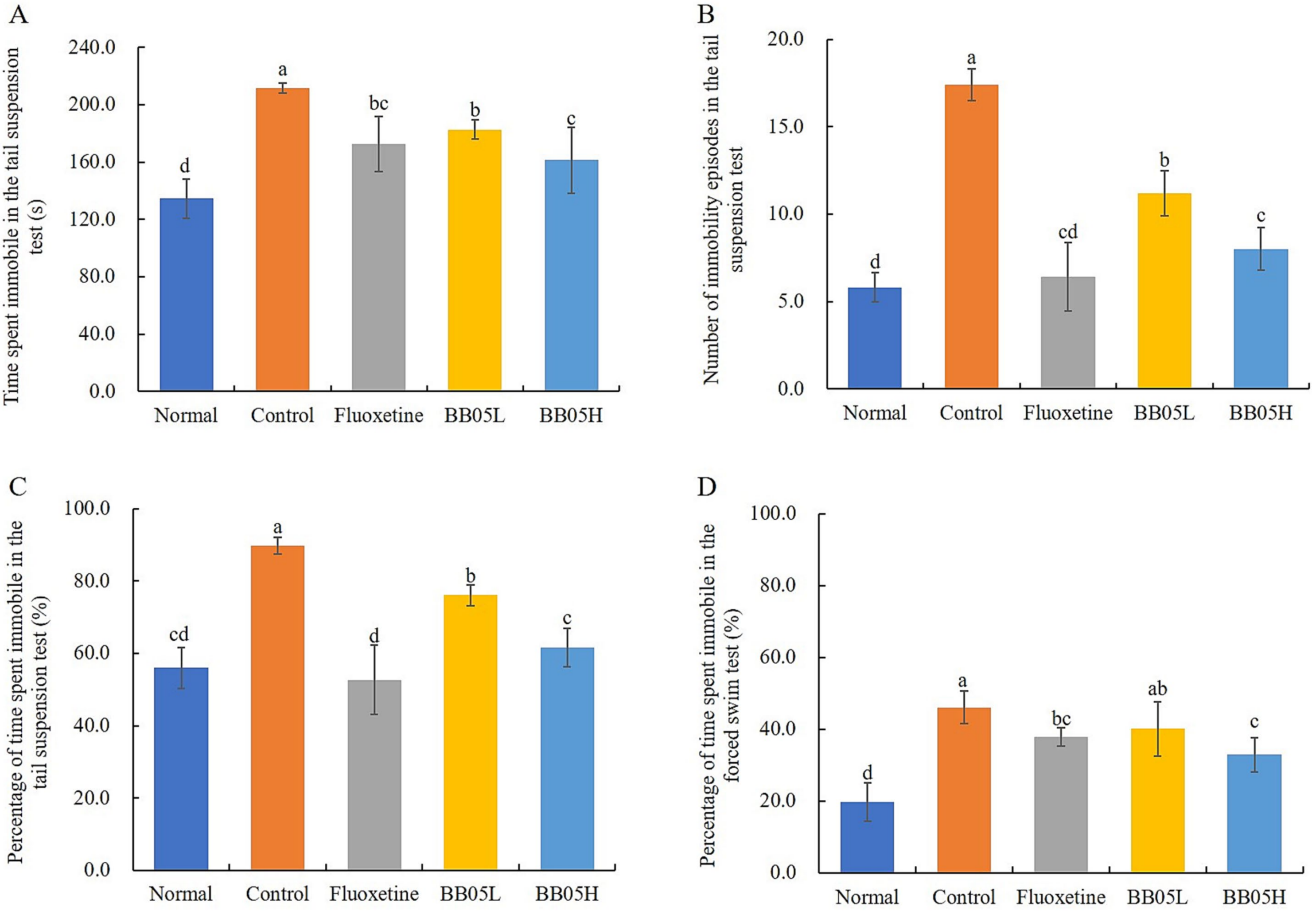
The effect of BB05 on tail suspension and forced swimming behaviors in mice. **(A)** Immobility time in the tail suspension test; **(B)** Number of immobility episodes in the tail suspension test; **(C)** Percentage of immobility time in the tail suspension test; **(D)** Proportion of immobility time in the forced swimming test (%). Different lowercase letters indicate significant differences (*p* < 0.05).

The tail suspension test and forced swimming test were used to assess depressive-like behaviors in animals, where immobility time is positively correlated with despair. As shown in Figure 3A, the normal group exhibited an immobility time of 134.42 ± 13.56 s, which significantly increased in the model group to 211.72 ± 3.49 s, indicating profound despair. After treatment with fluoxetine and BB05, the immobility time significantly decreased compared to the model group (*p* < 0.05). Notably, there was no significant difference in immobility time between the BB05H group and the fluoxetine group (*p* > 0.05). The same patterns were observed for the number of immobility episodes and the percentage of immobility time in the tail suspension test (Figures 3B,C).

Additionally, in the forced swimming test (Figure 3D), the proportion of immobility time was significantly higher in the model group compared to the normal, fluoxetine, and BB05H groups, further confirming the despair behavior in the model group. Fluoxetine and BB05 interventions effectively reduced immobility time, with the BB05H group showing greater efficacy than the BB05L group.

### 3.3 The effects of BB05 on the histopathology of mouse ileum tissue

**Figure 4:**
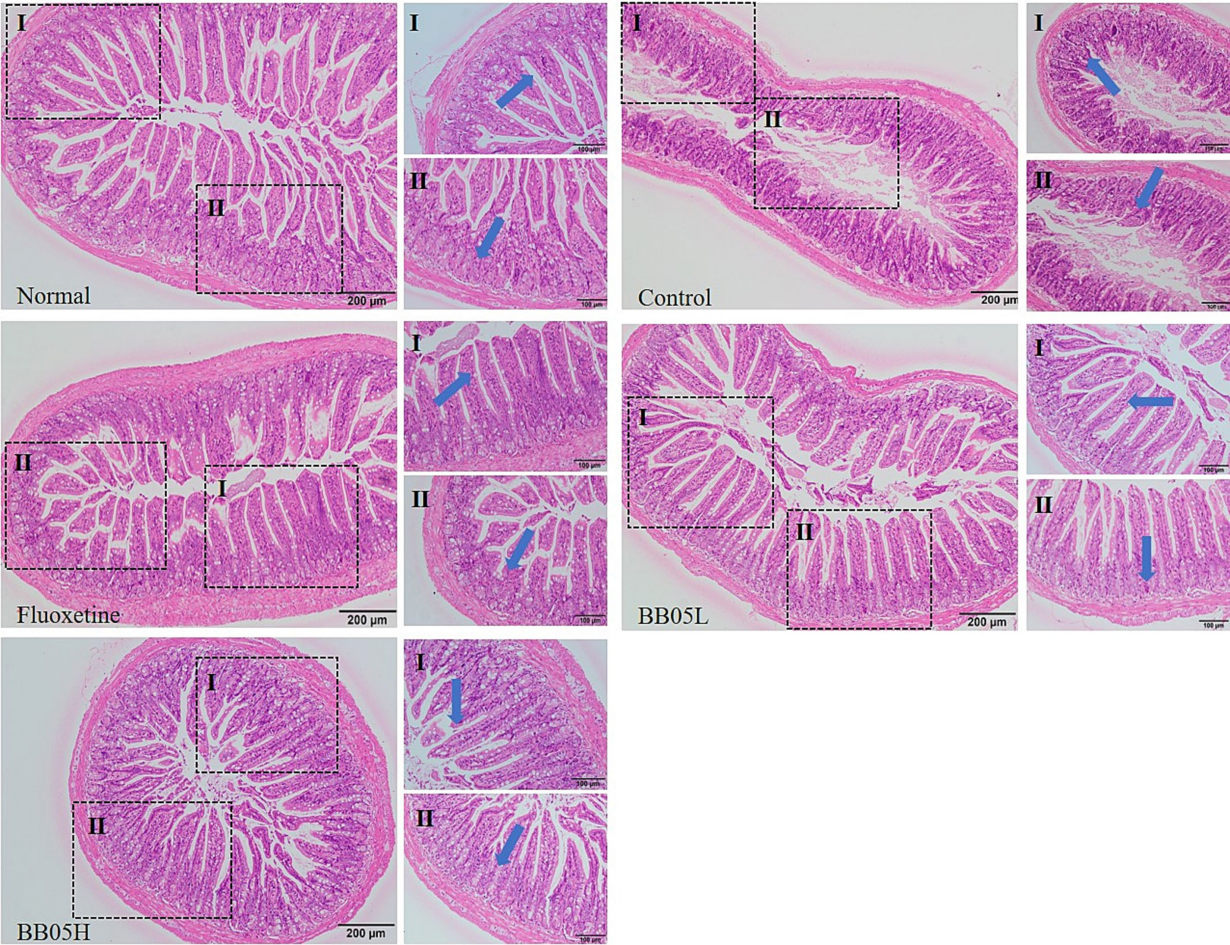
The effect of BB05 on the histopathology of the ileal tissue in mice. Note that the arrows in the figure indicate crypt structures (I) and whether or not they are damaged (II) in the ileal tissue.

As shown in Figure 4, the ileal tissue of mice in the control group displayed a well-organized and intact structure, with clearly defined layers and no signs of damage, inflammation, or swelling, indicative of a healthy state. In contrast, the model group exhibited disorganized ileal cells, abnormal crypt structure, significant infiltration of inflammatory cells, mucosal swelling, and areas of mucosal shedding. Treatment with fluoxetine and BB05 significantly alleviated these intestinal abnormalities. Mice in these groups showed restored intestinal structure, healthy crypt formation, organized villi, and an increased presence of goblet cells. Notably, severe pathological changes were not observed in any treatment group. However, the low-concentration BB05 group did show localized infiltration of inflammatory cells. These findings suggest that BB05 effectively mitigates pathological damage to the ileal tissue induced by chronic stress, with the high-concentration BB05 intervention exhibiting superior protective effects compared to the low-concentration group.

### 3.4 The effect of BB05 on the concentrations of 5-HT, ACTH, and IL-6 in mouse serum

**Figure 5:**
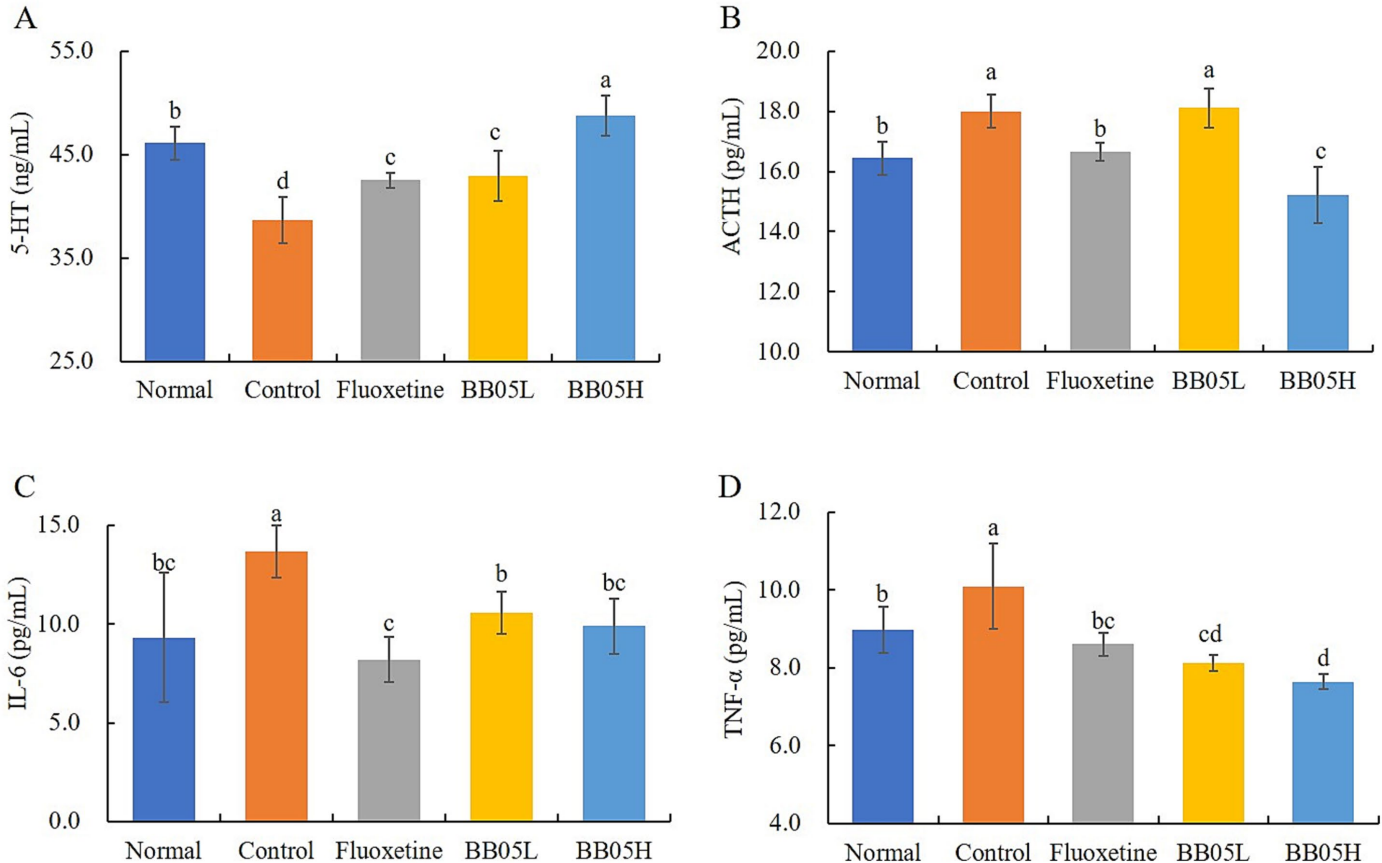
The effect of BB05 on serum 5-HT, ACTH, and IL-6 in mice. **(A)** Serum 5-HT concentration; **(B)** Serum ACTH concentration; **(C)** Serum IL-6 concentration; **(D)** Serum TNF-α concentration. Different lowercase letters indicate significant differences (*p* < 0.05).

5-HT is a neurotransmitter that plays a critical role in regulating mood and promoting pleasurable emotions, thereby potentially playing a role in depression induction. A reduction in 5-HT levels is closely associated with psychological changes and depression (40). Higher 5-HT concentrations are correlated with lower levels of depression. Figure 5A shows that the 5-HT level in the model group was the lowest, with a significant difference compared to all other groups (*p* < 0.05). Following fluoxetine and BB05 interventions, the serum 5-HT concentration in mice significantly increased (*p* < 0.05), with the BB05 high concentration group displaying higher levels than both the fluoxetine and BB05 low concentration groups. Concurrently, CUMS induced a significant increase in serum ACTH levels, as illustrated in Figure 5B. After fluoxetine and BB05 treatment, the CUMS-induced increase in ACTH levels was significantly reduced, with the BB05H group demonstrating superior results compared to the BB05L group (*p* < 0.05). Furthermore, the pro-inflammatory cytokines interleukin-6 (IL-6) and tumor necrosis factor-alpha (TNF-α) are critical regulators of inflammation and immunity. Figures 5C,D show that the IL-6 and TNF-α levels in the model group were significantly higher than in all other groups, indicating that chronic stress leads to elevated inflammation. Post-intervention with fluoxetine and BB05, serum concentrations of IL-6 and TNF-α significantly decreased (*p* < 0.05), demonstrating that both treatments effectively reduce inflammation, with the BB05H group showing greater efficacy than the BB05L group.

### 3.5 The effect of BB05 on the relative expression levels of AKT/mTOR in the prefrontal cortex of mice

**Figure 6:**
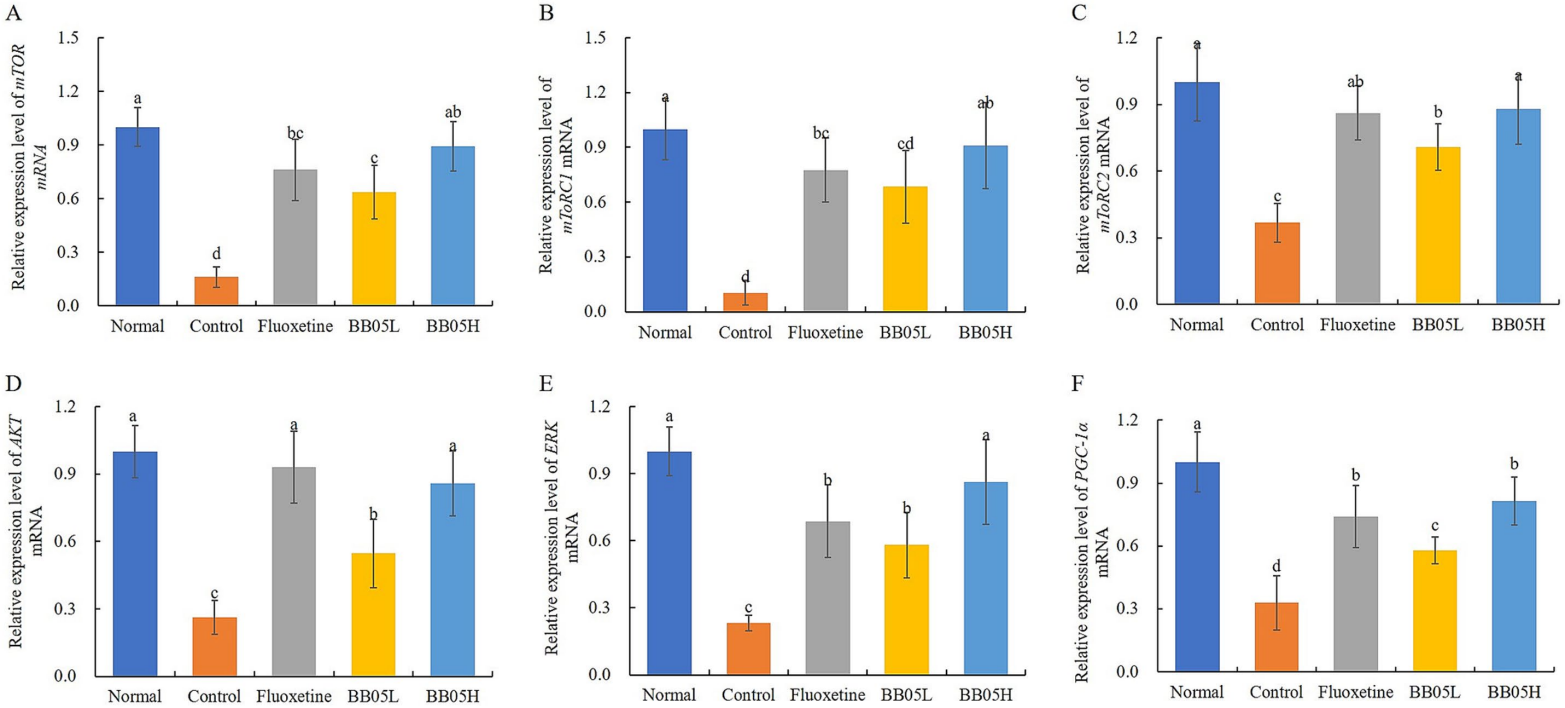
The effect of BB05 on the expression levels of mTOR pathway-related genes in the prefrontal cortex of the mouse brain. **(A)** Relative expression of mTOR; **(B)** Relative expression of mTORC1; **(C)** Relative expression of mTORC2; **(D)** Relative expression of AKT; **(E)** Relative expression of ERK; **(F)** Relative expression of PGC-1α. Different lowercase letters indicate significant differences (*p* < 0.05).

As shown in Figure 6, chronic stress significantly downregulates the expression of key components in the AKT/mTOR signaling pathway in the prefrontal cortex of mice. The expression levels of mTOR, mTORC1, mTORC2, AKT, ERK, and PGC-1α were significantly reduced in the model group compared to the control group (*p* < 0.05). However, fluoxetine and BB05 interventions restored the expression of these factors (*p* < 0.05). Notably, the BB05H group exhibited higher expression levels of mTOR, mTORC1, mTORC2, and ERK compared to the fluoxetine-treated group. These findings suggest that both fluoxetine and BB05 effectively normalize the expression of AKT/mTOR pathway-related factors, with the BB05H group showing superior results over the BB05L group.

## 4 Discussion

This study effectively utilized the CUMS model to induce depression-like behavior in mice, establishing a robust foundation for evaluating the antidepressant potential of *Bifidobacterium breve* BB05 (BB05). The CUMS model is widely recognized in depression research, as it replicates depression-like symptoms in animals through various unpredictable stressors, such as weight loss, reduced activity, and increased anxiety and depression-like behaviors. Our results demonstrated that mice subjected to CUMS exhibited significant weight loss and behavioral changes, validating the model’s reliability.

Following CUMS induction, mice showed a significant decrease in weight gain, consistent with the well-established link between chronic stress and appetite loss, leading to weight reduction (41). Notably, BB05 intervention, particularly at a higher dose (BB05H), significantly improved the rate of weight gain compared to the CUMS model group. This suggests that BB05 mitigates the harmful effects of chronic stress, aiding in weight recovery. The mechanism may involve BB05’s modulation of gut microbiota, potentially enhancing nutrient absorption and metabolism, thereby counteracting stress-induced metabolic disruptions (9).

Behavioral assessments, including the open field test, tail suspension test, and forced swim test, revealed significant improvements in anxiety and depression-like behaviors following BB05 treatment. Mice in the CUMS model group displayed pronounced anxiety and depressive behaviors across all tests. However, BB05 intervention significantly alleviated these behavioral abnormalities, with some improvements exceeding those observed in the fluoxetine-treated group. This suggests that BB05 may regulate central nervous system function through the gut-brain axis. Probiotics, including BB05, are known to influence neurotransmitter levels in the brain, thereby affecting mood and behavior (42). Possible mechanisms include BB05’s ability to modulate gut microbiota composition, produce short-chain fatty acids, and synthesize neuroactive substances, which, in turn, impact central nervous system activity (43).

The ileal tissue of mice in the CUMS group exhibited significant pathological damage, including disordered cell structure, inflammatory cell infiltration, and mucosal swelling. In contrast, treatment with BB05 notably improved the condition of the ileal tissue, especially in the high-dose group. These results suggest that BB05 effectively mitigates chronic stress-induced intestinal inflammation and damage, likely through its regulation of gut microbiota, enhancement of intestinal barrier function, and anti-inflammatory effects (44). Furthermore, BB05 treatment significantly increased serum 5-HT levels while reducing ACTH and pro-inflammatory markers such as IL-6 and TNF-α. This highlights BB05’s antidepressant and anti-inflammatory properties. Serotonin is a key neurotransmitter involved in mood regulation, with increased 5-HT levels potentially linked to BB05’s modulation of gut microbiota, which may enhance the production and transport of 5-HT precursors (45). ACTH, which regulates cortisol levels, plays a role in the emotional and cognitive symptoms of depression through its impact on the hypothalamic–pituitary–adrenal (HPA) axis (46). Elevated IL-6 and TNF-α levels, commonly observed in individuals with depression, contribute to inflammation and HPA axis activation, influencing mood regulation, neurotransmitter metabolism, and neural plasticity, thereby exacerbating depressive symptoms (47). The reduction in ACTH and pro-inflammatory markers observed in this study suggests that BB05 may alleviate chronic stress-induced endocrine and immune dysregulation by modulating the gut-brain axis, restoring neurotransmitter balance, and potentially relieving depressive symptoms.

This study also explored the mechanism by which BB05 exerts its antidepressant effects in the prefrontal cortex, focusing on the AKT/mTOR signaling pathway. CUMS significantly reduced the expression of AKT/mTOR-related factors in the prefrontal cortex. However, BB05 intervention, particularly at high doses, significantly increased the expression of these factors, even surpassing the effects of fluoxetine. The AKT/mTOR pathway is critical for neuroprotection, neurogenesis, and synaptic plasticity. Its activation enhances BDNF expression, promoting neurogenesis and neural repair, which contributes to its antidepressant effects (48). Upon activation, AKT undergoes phosphorylation at the cell membrane, promoting cell growth, metabolism, and survival by activating downstream pathways, including the direct phosphorylation of mTOR (49). mTOR functions in two distinct complexes: mTORC1, which regulates protein synthesis and cell growth, and mTORC2, which influences cell metabolism and survival (50). Additionally, the ERK pathway, known to regulate neural plasticity and survival, plays a role in depression, with abnormal ERK signaling associated with depressive symptoms (51). Another important factor, PGC-1α, regulates mitochondrial biogenesis and energy metabolism. Its decreased expression in depression may result in energy metabolism disorders and neuronal dysfunction, worsening depressive symptoms (52). BB05’s antidepressant effects may therefore be attributed to its activation of the AKT/mTOR pathway, enhancing neuroprotection and neural plasticity.

These findings align with existing literature, reinforcing the role of probiotics in managing depression. Multiple studies confirm that probiotics can modulate central nervous system function and alleviate depression and anxiety by regulating the gut-brain axis (53, 54). Previous research also shows that probiotics exhibit antidepressant and anxiolytic effects through various pathways, including neurotransmitter modulation, neurogenesis promotion, gut health improvement, and the reduction of inflammatory responses (55).

While this study highlights the significant antidepressant effects of BB05, several limitations require further investigation. First, as the research was conducted using a mouse model, clinical trials are needed to confirm the efficacy and safety of BB05 in humans. Second, the study primarily focused on behavioral and physiological indicators; more detailed molecular studies are necessary to uncover the specific mechanisms underlying BB05’s effects. It is important to note that a notable limitation of this study is the lack of conclusive evidence for AKT/mTOR pathway activation. Despite observing changes in mRNA expression levels, the Western blotting experiments, which are essential for directly detecting the activation status of key proteins in the pathway, were deficiency. As mRNA expression does not necessarily reflect protein activity or pathway activation, relying solely on the mRNA data presented here is insufficient to confirm the activation of the AKT/mTOR pathway. This limitation underscores the need for further investigation, and in future research, we will aim to optimize Western blotting techniques and potentially employ additional methods to more comprehensively validate the activation of this pathway. Additionally, while the research emphasized the AKT/mTOR signaling pathway, depression involves complex and diverse pathological mechanisms, warranting exploration of other potential pathways. Further studies should also examine the differential effects of various probiotic strains and dosages to optimize their therapeutic use for depression.

This study demonstrated that BB05H significantly alleviated depression-like behaviors induced by CUMS in mice and improved several physiological markers. Potential mechanisms include the regulation of the gut-brain axis, increased 5-HT levels, reduced pro-inflammatory factors, and activation of the AKT/mTOR signaling pathway. These findings suggest that probiotics may offer a novel and effective intervention for depression. However, further research is necessary to validate their clinical efficacy and safety, as well as to explore their mechanisms of action. This will provide a theoretical foundation for developing safer and more effective strategies for depression treatment.

## Data Availability

The original contributions presented in the study are included in the article/supplementary material, further inquiries can be directed to the corresponding authors.
